# Data of a triboelectric energy harvester in low frequency environment and its potential as a power source in a wireless sensor node

**DOI:** 10.1016/j.dib.2026.112670

**Published:** 2026-03-09

**Authors:** Marios Kaminiotis, Julien Le Scornec, Romain Noël, Vincent Le Cam, Bastien Chapuis

**Affiliations:** a I4S, COSYS-SII, Inria, Université Gustave Eiffel, F-44344 Bouguenais, France; b Université Paris-Saclay, CEA, LIST, F-91120, Palaiseau, France

**Keywords:** Vibration energy harvesting, Low frequency vibrations, Energy conversion, Structural health monitoring

## Abstract

The current article highlights the potential usage of a triboelectric nanogenerator (TENG) as a power source in a wireless sensor node in the field of Structural Health Monitoring (SHM). The voltage response of the TENG across different resistors and capacitors is used to characterize the power output of the vibration energy harvester and it estimates the energy that the TENG can provide to a circuitry. The provided data were collected using a custom-made LEGO platform, which was set to operate in low frequency sinusoidal motion, simulating a low frequency vibration environment of a bridge cable. This paper aims to encourage the usage of such effective energy harvesters in SHM by showcasing their potential in powering up a wireless sensor node directly, or act as a complementary energy source to a battery.

Specifications Table

**Table:** 

Subject	Engineering & Materials science
Specific subject area	Energy Harvesting, Structural Health Monitoring, Low Frequency Vibration
Type of data	Raw, Processed, Table (Comma-Separated Values (CSV) files, eXcel SpreadSheet (XLS) files).
Data collection	The generated output voltage of multiple triboelectric nanogenerators (TENGs) across various resistors was measured with a digital oscilloscope (LeCroy WaveRunner 104Xin1 GHz, Tektronics) utilizing a high impedance probe (Tektronics P5122). Current, power and energy per cycle were calculated through a home-developed python script. Additionally, the time to charge various capacitors connected in parallel with the TENG devices was measured using a NI USB-6211 box with 250 KS/s sampling rate and 16-bit sampling capacity.
Data source location	Université Gustave Eiffel Campus de Nantes, Nantes, France
Data accessibility	Repository name: Zenodo Data identification number: https://doi.org/10.5281/zenodo.18772195 Direct URL to data: https://zenodo.org/records/18772195
Related research article	None

## 1 Value of the Data

• The data will contribute to expand the scope of knowledge on triboelectric and vibrational energy harvesters, which are extensively studied by the scientific community for applications such as the Internet of Things (IoT) and wireless sensors.
• The results from this dataset could inspire new designs based on this energy harvesting topology, which can be adapted by the scientific community to address other research challenges.
• This dataset provides experimentally measured voltage output characteristics of multiple triboelectric nanogenerators (TENGs) under low-frequency excitation (1 - 4 Hz), representative of vibration profiles in civil infrastructure and in bridge environments. The data enable benchmarking of tribo-pairs based on experimental results rather than theoretical triboelectric series, extraction of internal impedance for impedance-matching circuits or power management circuits and quantitative estimation of the harvested energy in low-frequency environments.
• Researchers, electrical engineers, circuit designers, professionals and companies involved in bridge maintenance may benefit from this dataset as it supports validation of analytical and equivalent circuit TENG models, design of impedance-matching and power management circuits, assessment of energy-autonomous wireless sensor nodes for bridge monitoring applications. Additionally, the data enable early-stage device sizing and system-level energy budgeting.
• The data can be analyzed with the developed python script reported in this paper enabling comparison of the various TENGs in terms of voltage, current, power, energy per cycle. This facilitates univariate and bivariate performance evaluation across material pairs, device sizes and load conditions.

## 2 Background

Wireless Sensor Networks (WSNs) have found application in a wide range of everyday life aspects including smart homes [1,2], precision agriculture [3] and structural health monitoring (SHM) [4,5]. In SHM, assessing the structural integrity of civil engineering structures, such as bridges, is crucial public safety. These vast networks consist of many individual sensor nodes, generally powered up by batteries of limited lifetime, making the replacement of depleted batteries time-consuming and expensive [6]. Energy harvesting offers an attractive alternative option for powering up the sensor nodes directly or at least to act as a complementary energy source that will charge the battery, increasing its lifetime, hence reducing maintenance costs due to the increased time interval between the replacement of the battery. Typically, a sensor node is exposed in various environmental factors, such as the sun, the wind and vibrations [7]. In the sensor node's environment, the ambient vibrations are usually characterized by low frequency (<10 Hz) and low amplitude (up to 10 mm) [8]. Specifically, data from cable-stayed bridges show low natural frequencies and amplitudes ranging from sub-centimeter up to several centimeters [9]. This dataset investigates the power generation of a triboelectric nanogenerator (TENG) in this low frequency and amplitude environment utilizing a custom-made LEGO platform as a linear motor.

## 3 Data Description

The following section describes in full detail the dataset of different measurements to characterize the performance of a TENG as a vibration energy harvester that operates in a low frequency environment (1 - 4 Hz).

The dataset includes eight TENG devices of different sizes (3.5 × 3.5 cm^2^ and 5 × 5 cm^2^) and four material pairs (PTFE - Nylon 66, FEP - Nylon 66, PTFE - PVDF/HFP and FEP - PVDF/HFP). For each device, two different experiments were conducted in all reported frequencies (1 - 4 Hz), the resistor sweep and the capacitor charging. In the Resistor sweep experiments, the voltage output across different resistors (1 - 200 MΩ) was measured to identify the resistance value where the maximum energy generation occurs. In addition, in the capacitor charging test, the voltage across different capacitors (1 and 10 µF) was measured to record how quickly the TENG can charge it and make the stored energy available for later use. Below there is a Quick-Guide to the dataset:

• Devices: 8 total (2 sizes: 3.5 × 3.5 and 5 × 5 cm^2^, 4 pairs (PTFE-Nylon 66, PTFE-PVDF/HFP, FEP-Nylon 66, FEP-PVDF/HFP)
• Resistor Sweep (.csv): 16 loads (1 - 200 MΩ, time measured in seconds)
• Capacitor Charge (.xls): 2 loads (1 and 10 µF, time measured in milliseconds)
• Test condition: 1, 2, 3, 4 Hz at 1 cm amplitude.

In the repository two main folders exist: the **Data_Paper** folder which contains the processed data and the **Data_Paper_Raw** folder which contains the raw data [10]. Both of them are organized hierarchically by device size, conducted experiment, triboelectric pair and external vibration frequency. Users should note that Resistor sweep files are in CSV form and the time is measured in seconds (s), meanwhile the capacitor charging files are in XLS form and the time is measured in milliseconds (ms).

The **Data_Paper** folder contains two subfolders named by the dimensions of each device **3.5** **×** **3.5** and **5** **×** **5**. Each one of those folders contain additional subfolders named after the particular experiments, **Resistor_Sweep** and **Capacitor_Charge**. The data generated from the measurements on various resistors and capacitors are in CSV and XLS form, respectively.

In each **Resistor_Sweep** subfolder there are four subfolders named after each individual device, such as **FEP – Nylon, FEP – PVDF-HFP, PTFE – Nylon, PTFE – PVDF-HFP**. In each one of those folders there are subfolders corresponding to the connected resistor, namely **1 Mohm, 10 Mohm, 20 Mohm, 30 Mohm, 40 Mohm, 50 Mohm, 60 Mohm, 70 Mohm, 80 Mohm, 90 Mohm, 100 Mohm, 127 Mohm, 154 Mohm, 181 Mohm, 200 Mohm** and **Voc.** Inside those subfolders, there are four files that correspond to the four different frequencies tested, namely **1** **Hz.csv, 2** **Hz.csv, 3** **Hz.csv, 4** **Hz.csv**. In each CSV file there are two columns, **Time (s)** and **Voltage (V).**

In each **Capacitor_Charge** subfolder, there are four subfolders named after each individual device, such as **FEP – Nylon, FEP – PVDF-HFP, PTFE – Nylon, PTFE – PVDF-HFP**. In each one of those folders there are subfolders corresponding to the connected capacitor, namely **1 uF** and **10 uF.** Inside those subfolders, there are four files that correspond to the four different frequencies tested, namely **1** **Hz.xls, 2** **Hz.xls, 3** **Hz.xls, 4** **Hz.xls**. In each XLS file there are two columns, **Time (ms)** and **Voltage (V).**

**Fig. 1:**
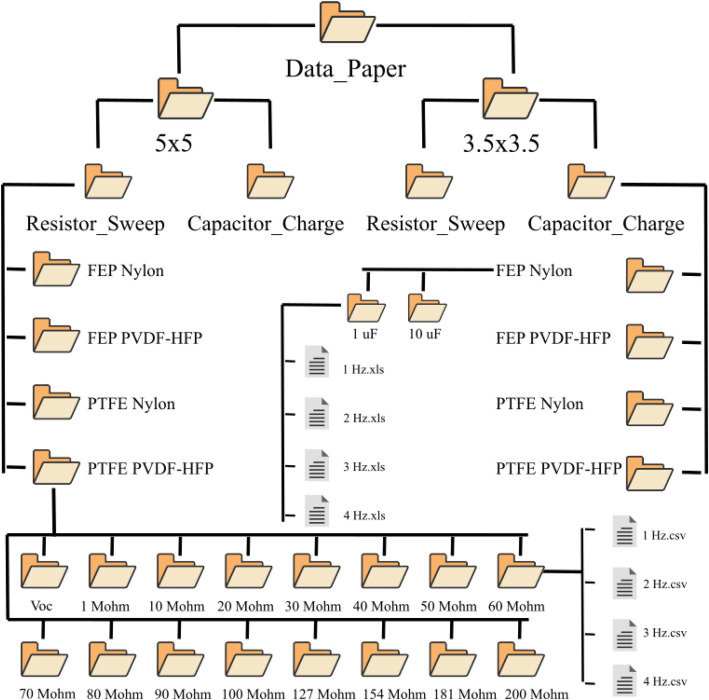
Structure and description of the Data_Paper folder.

In Fig. 1, there is a schematic illustration of the above-described data paths in two examples. In particular, the **FEP-Nylon** device was selected from the **3.5** **×** **3.5** folder to showcase the subfolders in the **Capacitor_Charge** folder and the **PTFE – PVDF HFP** device was selected from the **5** **×** **5** folder to showcase the subfolders in the **Resistor_Sweep** folder.

**Fig. 2:**
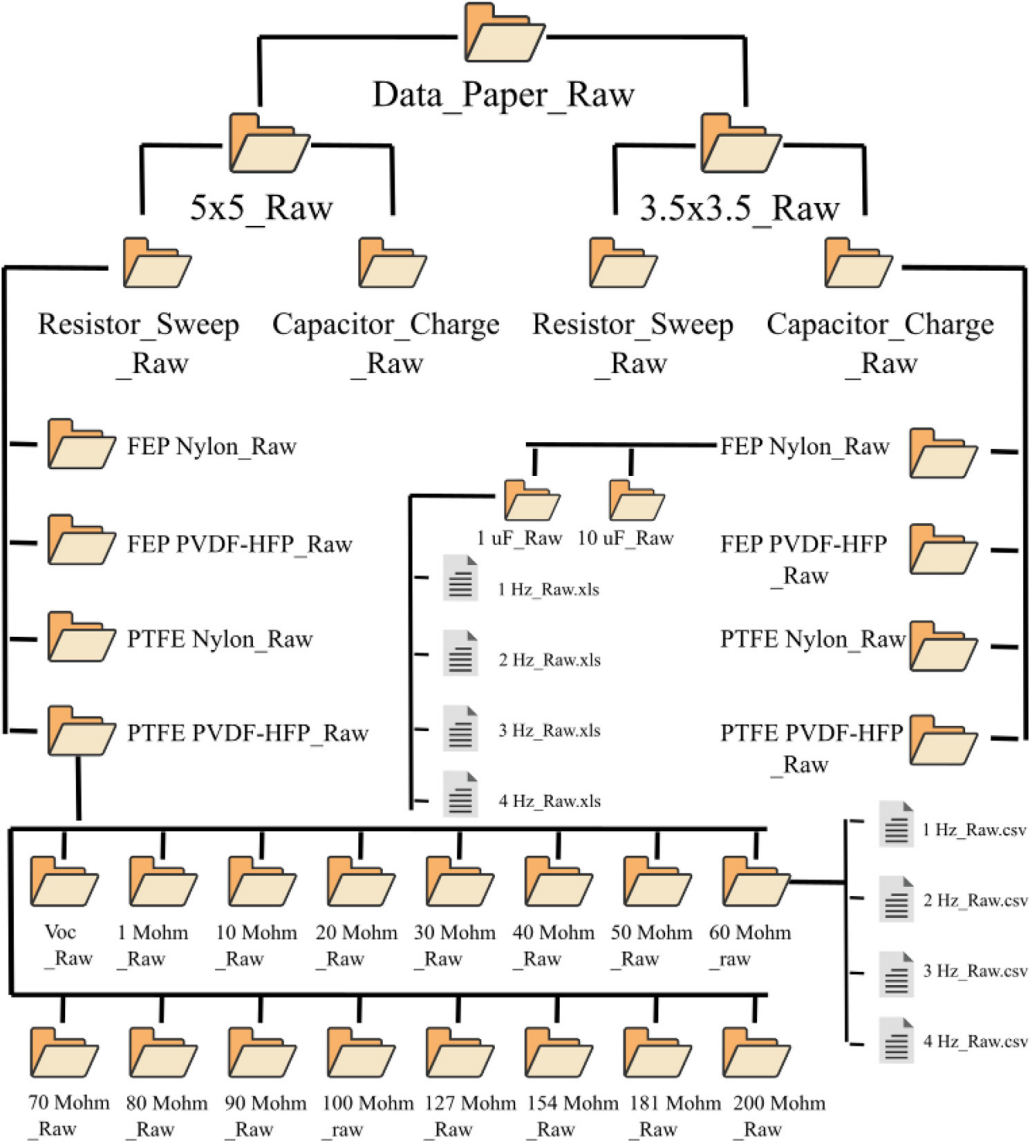
Structure and description of the Data_Paper_Raw folder.

The **Data_Paper_Raw** folder follows the same directory structure as the **Data_Paper** folder. The only difference between them is that the suffix**_Raw** is added to each folder and file name. The structure of the **Data_Paper_Raw** folder is show in Fig. 2.

## 4 Experimental Design, Materials and Methods

The triboelectric materials that were selected were Polytetrafluoroethylene (PTFE), Polyamide 66 (Nylon 66), Fluorinated Ethylene Propylene (FEP), and Poly (vinylidene fluoride-co-hexafluoropropylene) (PVDF/HFP) and they were purchased from Sigma Aldrich. Double sided copper tape purchased from 3 M Mouser Electronics was used as an electrode in every device. A total of eight devices were fabricated, measured, and characterized.

For the fabrication of those devices, two acrylic plates of 3.5 × 3.5 cm^2^ and 5 × 5 cm^2^ respectively were used as substrates to deposit the triboelectric materials and their respective electrodes. For each substrate size four different triboelectric pairs were selected (FEP – Nylon, FEP – PVDF/HFP, PTFE – Nylon and PTFE – PVDF/HFP). The dimensions of each device are listed in Table 1.

**Table 1:** Materials and their dimensions of each fabricated TENG device.

Device Name	Tribonegative Material	Dimensions (cm x cm x µm)	Tribopositive Material	Dimensions (cm x cm x µm)	Electrodes
***TENG1***	*PTFE*	*5×5×25*	*Nylon 66*	*5×5×17*	*Double Sided Copper Tape*
***TENG2***	*PTFE*	*3.5×3.5×25*	*Nylon 66*	*3.5×3.5×17*	*Double Sided Copper Tape*
***TENG3***	*FEP*	*5×5×25*	*Nylon 66*	*5×5×17*	*Double Sided Copper Tape*
***TENG4***	*FEP*	*3.5×3.5×25*	*Nylon 66*	*3.5×3.5×17*	*Double Sided Copper Tape*
***TENG5***	*PTFE*	*5×5×25*	*PVDF/HFP*	*5×5×500*	*Double Sided Copper Tape*
***TENG6***	*PTFE*	*3.5×3.5×25*	*PVDF/HFP*	*3.5×3.5×500*	*Double Sided Copper Tape*
***TENG7***	*FEP*	*5×5×25*	*PVDF/HFP*	*5×5×500*	*Double Sided Copper Tape*
***TENG8***	*FEP*	*3.5×3.5×25*	*PVDF/HFP*	*3.5×3.5×500*	*Double Sided Copper Tape*

**Fig. 3:**
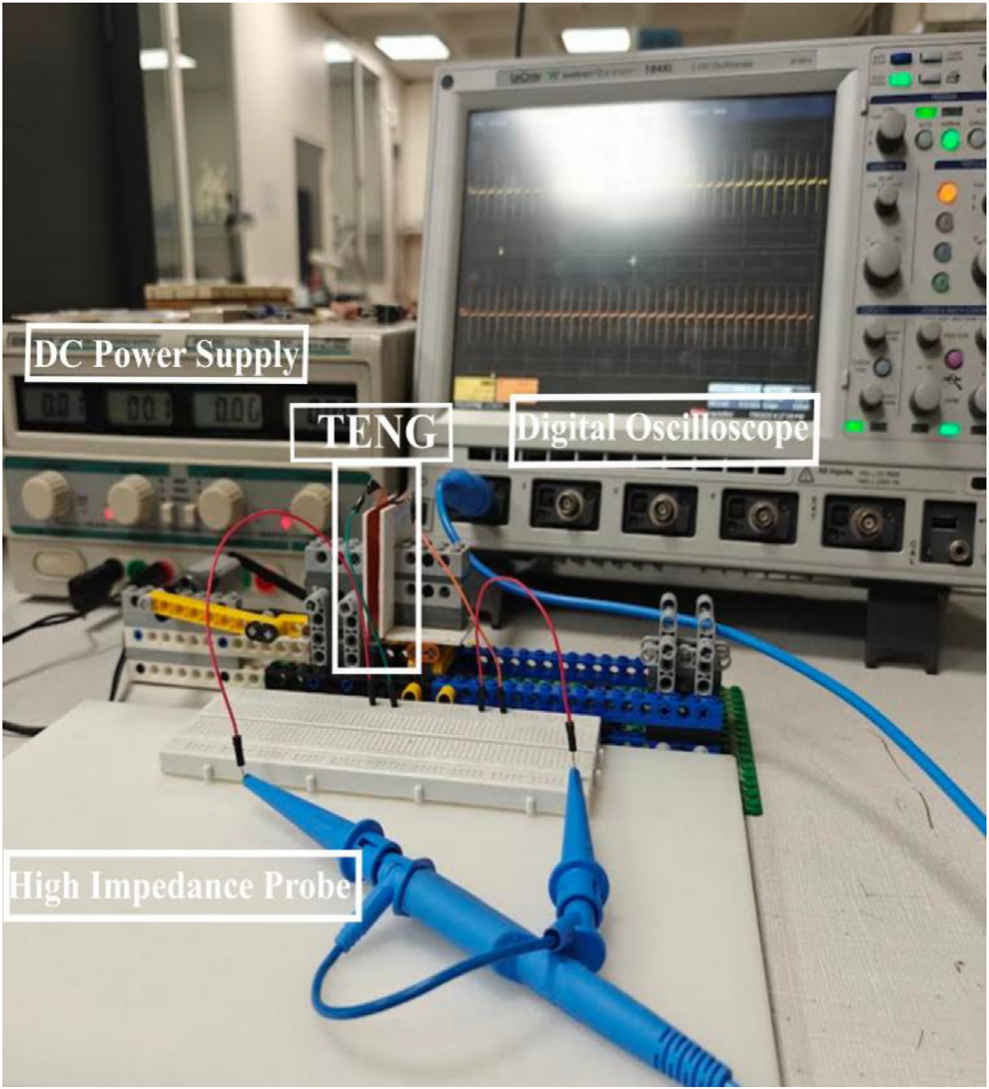
Home-made experimental LEGO setup.

A custom-made LEGO setup was used as a linear motor to characterize the output performance of various TENG devices. The system consisted of a LEGO motor, driven by a DC voltage supplier (Vellman LABPS23023, 0 - 30 V/ 0–3 A, x2WAY), which was able to move a LEGO platform in the lateral direction with an amplitude of A_0_ = 1 cm in a range of frequencies (1, 2, 3 and 4 Hz). The circular arm that connects the LEGO motor with the LEGO platform rotates with radius of 0.5 cm. When it is at the top point the two tribomaterials are touching. When it is at the lower point of its movement the tribomaterials have the maximum separation distance. This translates to lateral distance of 1 cm. This rotational movement restricts the LEGO platform to the fixed amplitude of 1 cm. The oscillation frequency was adjusted by repeatedly measuring the signal’s period of oscillations in a large time window in the oscilloscope and then adjusting the voltage to achieve the desired frequencies of 1 Hz, 2 Hz, 3 Hz and 4 Hz. More specifically, the voltage displayed on the DC power supplier was 2.3 V, 3.3 V, 4.3 V and 5.3 V for 1 Hz, 2 Hz, 3 Hz and 4 Hz respectively. The validation of the proper frequency was done before and after each measurement to ensure consistency throughout the experiments. Since the motion is sinusoidal and the mass of the LEGO tower doesn't change for the different tribomaterials, under the same frequency conditions the contact force should be similar. The alignment of the materials was manually verified before every experimental run to secure an appropriate contact-separation cycle, where the surface area of both materials overlapped during contact. Its unique design to perform the particular movement allowed the simulation of a low-frequency vibration of a stay cable in a bridge. At the end of the LEGO platform, a LEGO tower was added. The corresponding tribo-negative layer of each TENG device was fixed firmly on the LEGO tower, meanwhile the corresponding tribo-positive layer was fixed at the end of the LEGO platform, as seen in Fig. 3.

The characterization of each energy harvester was conducted by connecting each device with various load resistors (1, 10, 20, 30, 40, 50, 60, 70, 80, 90, 100, 127, 154, 181, 200 MΩ and open-circuit -*R* = ∞) and various capacitors (1 and 10 µF) in different frequencies (1, 2, 3 and 4 Hz).

In particular, the generated voltage across the different resistors was measured by utilizing a high-impedance probe (P5122, Tektronix, Attenuation 100x, Bandwidth 200 MHz, Loading 100 MΩ/ 4 pF) and an oscilloscope (LeCroy WaveRunner 104Xi 1 GHz, vertical accuracy ± 1.5 % of the full-scale range) at a sampling rate of 25 kS/s. The positive end of the probe was connected to the tribopositive material and the other end of the probe was connected to the tribonegative material each time. The measuring window was 5 s capturing between 5 and 20 cycles, depending on the external frequency that was used. No digital filtering or averaging was applied during acquisition. Following the data collection, a custom Python script was developed for data analysis [11]. The script imports the measured voltage-time data from the CSV files and calculates the corresponding current and instantaneous power utilizing Ohm’s and Joule’s law, respectively:

(1) $$I\;=\;\frac{V}{R}$$

(2) P=V·I

Additionally, it identifies positive and negative peaks in voltage, and current and using height-threshold and a minimum distance (between peaks) algorithm. Then it computes the average peak-to-peak values of voltage and current by averaging the absolute values of the detected positive and negative peaks:

(3) $$V\;=\;\frac{\sum \left|V_{pos}\right|+\;\sum \left|V_{neg}\right|}{N_{peaks}}$$

(4) $$I\;=\;\frac{\sum \left|I_{pos}\right|+\;\sum \left|I_{neg}\right|}{N_{peaks}}$$

The power peaks were identified with the same algorithm as in voltage and current peaks. The average power was calculated by summing the magnitudes of positive peaks divided by half of the number of the detected peaks, reflecting the peak-to-peak power generated in a contact-separation event:

(5) $$P\;=\;\frac{\sum \left|P_{pos}\right|}{2N_{peaks}}$$

Furthermore, the area under each power peak is integrated in order to calculate the energy delivered to the resistive load using the trapezoidal rule:

(6) $$E\;=\;\int_{t_{start}}^{t_{end}}P\;\left(t\right)dt\;\approx \;\sum_{i}\frac{P\left(t_{i}\right)+\;P\left(t_{i+1}\right)}{2}\Delta t$$

The integration window was dynamically selected. A relative height of 98 % from the maximum peak was selected to define t_start_ and t_end_. Each pair of consecutive power peaks represents the contact and separation process of the TENG’s operation cycle. The energy per cycle is calculated by summing the energy of those two consecutive peaks:

(7) $$E_{cycle}\;=\;E_{peak-1}\;+\;E_{peak-2}$$

The script is able to calculate the average energy per contact-separation by averaging the energy in all the detected cycles:

(8) $$E=\frac{1}{n}\;\sum_{j=1}^{n}E_{j,cycle}$$

**Fig. 4:**
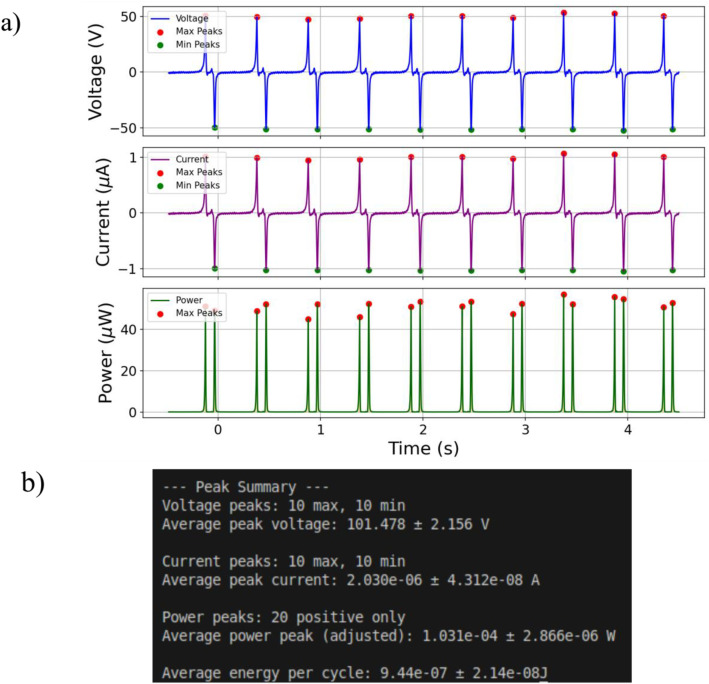
Data_Paper/5 × 5/Resistor_Sweep/PTFE Nylon/50 Mohm/2 Hz.csv: a) Illustration of voltage-time (blue) data, current-time (magenta), power-time (green) data, b) user’s terminal showcases all extracted values along with their standard deviation after running the script.

Data collection on those values allows the observation of the response of each device while increasing the external resistance, which it will determine the system’s matching impedance conditions. Finally, a summary of the results is printed and the voltage, current and power in respect to time are visualized. A representative example is illustrated in Fig. 4. For the data file: Data_Paper/5 × 5/Resistor_Sweep/PTFE Nylon/50 MOhm/2 Hz.csv, the peak-to-peak voltage, current, power and the energy per cycle along with their corresponding standard deviations were calculated. In particular, the reported values are: 101.478 ± 2.156 V, 2.030 ± 0.04312 μA, 103.1 ± 2.866 μW and 944 ± 21.4 nJ, respectively. These values correspond to a coefficient of variation (standarddeviation/peakvaluex100%) of 2.12 %, 2.12 %, 2.21 % and 2.27 % for voltage, current, power and energy per cycle, respectively, showcasing the repeatability of the experiments. In the open-circuit case, the same method is used, but the current and power peaks are ignored, focusing only on peak-to-peak voltage values.

A performance comparison between all the devices was conducted under selected and identical conditions. The highest output in all frequencies was demonstrated by TENG1 (size: 25 cm^2^ and consisting of PTFE-Nylon) and its output performance is illustrated in Table 2.

**Table 2:** Identification of best performing device under identical conditions.The data paths for the devices displayed in the following table are: Data_Paper/5 × 5/Resistor_Sweep/PTFE Nylon/100 Mohm/1 Hz.csv, Data_Paper/5 × 5/Resistor_Sweep/PTFE Nylon/100 Mohm/2 Hz.csv, Data_Paper/5 × 5/Resistor_Sweep/PTFE Nylon/100 Mohm/3 Hz.csv, Data_Paper/5 × 5/Resistor_Sweep/PTFE Nylon/100 Mohm/4 Hz.csv.

Frequency (Hz)	Load Resistance (MΩ)	Device Name	Voltage (V)	Current (μA)	Energy Per CS (μJ)	Power Density($\text{mW}\;/\;m^{2}$)
1	100	TENG1	112.19	1.12	*0.92*	*26.5*
2	100	TENG1	182.28	1.82	*1.78*	*71.24*
3	100	TENG1	229.94	2.3	*2.19*	*110.48*
4	100	TENG1	*245.97*	2.46	*2.42*	*128*

Regarding the capacitor charging measurements, the corresponding TENG was connected to a bridge rectifier (Vishay VS-1KAB80E - forward drop = 1.1 V per diode) which allowed the charging of a connected capacitor. The voltage-time data were acquired using a National Instrument box (NI USB-6211 Box, absolute accuracy: ± 1.5 mV) at the sampling speed of 250 kS/s and sampling capacity of 16-bit. The NI USB-6211 Box is controlled by a LabView Virtual Instrument (VI), which upon initialization generates a time-stamped XLS file and records voltage measurements at each sampling interval and it transmits the data via USB. A custom Python script was developed to import the data from the XLS file, remove initial abnormalities (due to improper grounding), and reset the time so the charging process begins at *t* = 0 s. Initial abnormalities refer to voltage fluctuation due to manual discharge of the capacitor utilizing metallic tweezers. Those fluctuations were removed from the uploaded processed files because they don’t represent TENGs’ charging performance. The script is able to identify the maximum voltage of 5.445 V and record the corresponding time. The value of maximum voltage and the corresponding time appear on the user’s terminal and a plot of the voltage in respect to time showcase the charging process of the capacitor. It should be noted that the 5.445 V threshold is the maximum measurable voltage allowed by the NI USB-6211 Box. This represents a limitation of the measuring system and not an upper limit of the TENG.

**Fig. 5:**
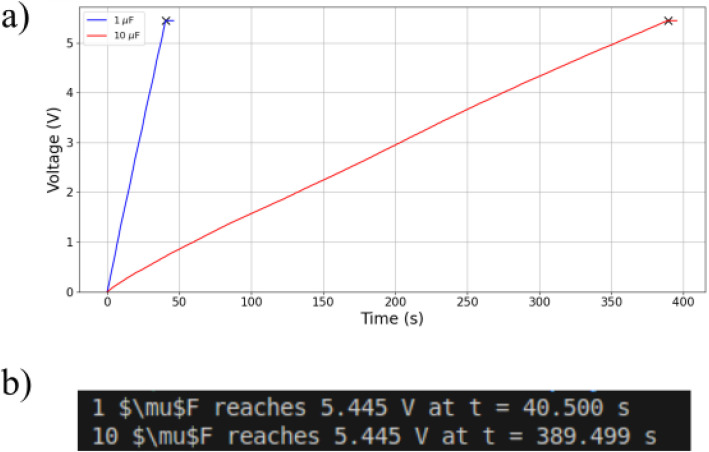
a) Charging process of two different capacitors 1 uF (blue - Data_Paper/5 × 5/Capacitor_Charge/PTFE Nylon/1 uF/2 Hz.xls) and 10 uF (red - Data_Paper/5 × 5/Capacitor_Charge/PTFE Nylon/10 uF/2 Hz.xls) by TENG1 under 2 Hz external vibration, b) user’s terminal showcase their respective time to reach the maximum voltage of 5.445 V.

Fig. 5 illustrates the processed data of the files: Data_Paper/5 × 5/Capacitor_Charge/PTFE Nylon/1 uF/2 Hz.xls and Data_Paper/5 × 5/Capacitor_Charge/PTFE Nylon/10 uF/2 Hz.xls. The charging curve of TENG1 can be observed along with the respective time to charge a 1 μF and a 10 μF capacitor.

Typically wireless sensor nodes have an operating voltage of 2.7–3.3 V [6]. Therefore, demonstrating that the TENG can charge a capacitor to a voltage above 3.3 V indicates that the harvested energy could supply low power wireless sensor nodes. Assuming that a sensor node is consisting of a temperature and humidity sensor (Bosch BEM280 [12]) and a system on chip (Nordic nRF52832 [13]). The energy consumed for a single event is calculated at approximately 83 μJ, using the sensor's data-sheet and the online power profiler [14]. Depending on the event frequency, the TENG could be power up directly this hypothetical sensor node (e.g., one event per hour) or act as a complementary power source to a battery by supplying energy for a single event once the capacitor is charged, thereby reducing battery usage.

## Limitations

The integration of a low-cost LEGO setup as a linear motor can provide a protocol to evaluate the performance of a TENG operating in contact-separation mode. Given the nature of the setup, the LEGO tower is not attached firmly on the LEGO platform which may leads to not optimal contact-separation cycles. The latter will affect the performance of the energy harvester negatively. Despite this mechanical constrain the observed variation of each electrical parameter was under 2.3 % for all electrical parameters, indicating that the LEGO setup is capable of providing reliable data.

Although tests were made in typical lab-environment conditions, environmental factors can affect the performance of TENGs. High humidity and very high temperature environments will negatively impact the output of the energy harvesters [15,16].

Regarding the capacitors charging measurements, the NI USB-6211 Box has a maximum measurable voltage of 5.445 V. By employing another NI USB Box with higher voltage threshold, the maximum voltage that a capacitor can reach while is charging from a TENG could be reported.

## Ethics Statement

The authors have read and follow the ethical requirements for publication in Data in Brief and confirming that the current work does not involve human subjects, animal experiments, or any data collected from social media platforms.

## CRediT authorship contribution statement

**Marios Kaminiotis:** Conceptualization, Validation, Investigation, Data curation, Writing – original draft, Writing – review & editing, Visualization. **Julien Le Scornec:** Validation, Data curation, Writing – review & editing, Supervision, Project administration, Funding acquisition. **Romain Noël:** Conceptualization, Validation, Resources, Writing – review & editing, Supervision, Project administration, Funding acquisition. **Vincent Le Cam:** Conceptualization, Validation, Resources, Writing – review & editing, Supervision, Project administration, Funding acquisition. **Bastien Chapuis:** Conceptualization, Validation, Resources, Writing – review & editing, Supervision, Project administration, Funding acquisition.

## Data Availability

ZenodoData of a triboelectric energy harvester in low frequency environment and its potential as a power source in a wireless sensor node. (Original data).
